# Molecular characterization of porcine circovirus 2 isolated from diseased pigs co-infected with porcine reproductive and respiratory syndrome virus

**DOI:** 10.1186/1743-422X-7-286

**Published:** 2010-10-27

**Authors:** Jianzhong Yi, Chengqian Liu

**Affiliations:** 1 Institute of Animal Husbandry Veterinary Sciences, Shanghai Academy of Agricultural Sciences, 2901 Beidi Road, Shanghai 201106, PR China

## Abstract

In this study, we isolated a porcine circovirus 2 (PCV2) strain from piglets co-infected with porcine reproductive and respiratory syndrome virus (PRRSV). The complete genome of this strain was sequenced, phylogenetic and polymorphic analyses were carried out. BLAST searches revealed the highest sequence identity (99.5% nt and 99.3% aa) to Guangxi strain EF675230. The phylogenetic tree showed that clustering of the isolates didn't strongly correlate to geographical distribution. Polymorphic analyses demonstrated that the amino acids at most of the polymorphic sites in Open Reading Frame 1(ORF1) and 2 (ORF2)belong to the same amino acid group according to chemical or structural properties, and revealed that highly polymorphic regions overlapped with the known immunoreactive epitopes of ORF2.

## 1 Introduction

Postweaning multisystemic wasting syndrome (PMWS), characterized by growth retardation, paleness of the skin, dyspnea, and increased mortality rates [1,2], was first described in Canada in 1991, and is now widespread throughout swine production areas of the world [3,4]. The genome of PCV is a single-stranded circular DNA of about 1.76 kb. ORF1 encodes the Rep proteins involved in virus replication and is highly conserved among isolates [5]. ORF2 encodes a 234 amino acid (aa) Cap protein, which is the main structural protein and also the major antigen inducing neutralizing immune responses [6]. The ORF3 protein is involved in PCV2-induced apoptosis by the caspase-8 and caspase-3 pathways [7]. However, healthy pigs experimentally inoculated with PCV2 developed only mild clinical symptoms [8,9], suggesting that other concomitant factors may be needed for the development of typical clinical PMWS [10,11]. Experimental studies on co-infection with PRRSV and PCV2 resulted in the microscopic lesions associated with PMWS and/or porcine dermatitis and nephropathy syndrome (PDNS), and lead to the development of severe disease [12].

**Figure 1:**
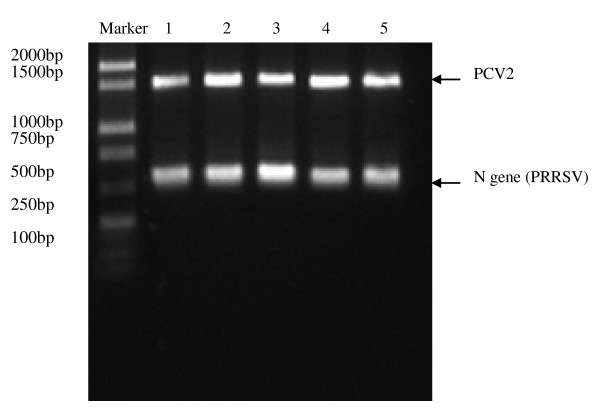
**Dection of PCV2 and PRRSV in five diseased pigs**. Blood samples from five diseased pigs were collected, viruses DNA and RNA were purified, respectively. After RT-PCR and PCR amplification, the amplified products run 1% agrose gel.

In may 2008, severe disease, known as ''high fever'' occurred in several pig farms in shanghai, leading to a 57% death rate. PRRSV was detected in all the diseased piglets, genome sequence blast showed the strain belongs to genotype 2, 99.4% homologous to the PRRS virus strain JXA1 isolated in China, which has been proved to cause porcine high fever disease with high morbidity and mortality[13]. There was no Porcine Parvovirus (PPV) detected in all the samples, but we detected PCV2 from all the PRRS infected piglets (Figure 1). To investigate the genetic relationship of this newly identified Shanghai PCV2 isolate with existing viruses isolated from other parts of the world, we sequenced the complete genome of the sh0901 strain and carried out phylogenetic and polymorphic analyses.

## 2. Materials and methods

### 2.1 Primer design and synthesis

Two pairs of primers were designed according to the published PCV2 genome sequence of strain AY691679, using the Primer 5.0 software. The sequences of the primers were as follows: upper primers, F1- 5'-AGAATTCAACCTTAACCTTTCT-3' and F2- 5'-AATCCTTCCGAAGACGAGCGCA-3'; reverse primers, R1- 5'-AGAATTCTGGCCCTGCTCC-3' and R2- 5'-TGGGCTCCACTGCTGTTATTC-3'. Primers were synthesized by the Shanghai Sangon Biological Engineering Technology & Services Co. Ltd. (Shanghai, PR China).

### 2.2 DNA extraction

Five homogenized Lung samples were collected from deceased pigs, DNA was extracted using a DNA mini kit (Tiangen Inc., PR China) according to the manufacturer's instructions.

### 2.3 Whole genome amplification

The thermocycling conditions were: 3 min at 94°C, then 35 cycles consisting of 30 s at 94°C, 35 s at 60°C and 2 min at 72°C, with a final extension at 72°C for 10 min. The PCR products were subjected to 1% (w/v) agarose gel electrophoresis, and visualized by ethidium bromide staining under UV illumination. Five PCR products were then subjected to DNA sequencing by the Shanghai Generay Biotech Co. Ltd. (Shanghai, PR China) with the same primers used for PCR.

### 2.4 Phylogenetic analysis of ORF2

Sequence comparisons were made by aligning the sequence of the virus isolate with those of other isolates using the algorithm CLUSTALW (version 1.8) method in the program MEGALIGN (DNASTAR, Lasergene version 7) at both the nucleotide (nt) and deduced aa levels. To analyze the homology and evolutionary relatedness, prototype genes for PCV2 were obtained from GenBank at the National Center for Biotechnology Information, USA (http://www.ncbi.nlm.nih.gov). Phylogenetic analyses were conducted using MEGA version 4.0 and elaborated with both parsimony and distance methods, supplying statistical support with bootstrapping over 1000 replicates.

## 3. Results

### 3.1 Genome sequence of the isolated strain

PCR amplification showed the five diseased pigs co-infected with PCV2 and PRRSV (Figure 1). The genome of the isolated viruses was 1767 nucleotide (nt) in length. The genome sequence was assembled, aligned using Seqman software (DNASTAR, Lasergene version 7), submitted to GenBank and assigned the accession number, GU124593. The genome sequence was confirmed to be PCV2 by BLAST searches against the GenBank database.

### 3.2 Sequence and phylogenetic analysis of ORF2

The ORF2 DNA sequence of the virus isolate shared 99.4% nt identity with the Guangxi isolate (accession number EF675230) and 94.0% with the Jiangsu isolate (accession number AY691679). The results indicated that the ORF2 sequence was not distinct in different geographical areas.

**Figure 2:**
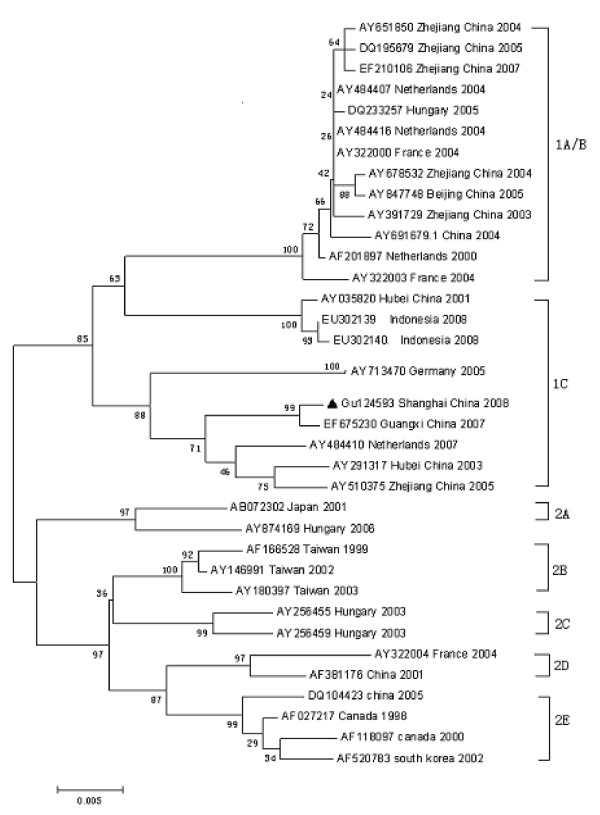
**Phylogenetic tree of the ORF2 gene of PCV2 strains**.

To determine the evolutionary relatedness of the new isolate, the ORF2 gene sequence was aligned with selected PCV2 isolates acquired from GenBank and performed phylogenetic analysis. The data showed that the virus isolate was classified into group 1 cluster C with the Guangxi strain (Figure 2).

### 3.3 Polymorphic analysis of ORF1, ORF2, and ORF3

Sequence alignment of the 46 geographically distinct PCV2 isolates revealed four polymorphic aa sites within ORF1 (Table 1). The aa at positions 35 are acidic residues, the aa at positions 77 and 105 belong to the group of nonpolar aa, while the aa at position 121 belong to the polar, uncharged group. Among the 22 polymorphic sites in ORF2, there were two aa differences at 16 sites, among which each pair of aa at the 12 sites belonged to the same amino acids group according to their chemical structure. There are six polymorphic sites in ORF3, the amino acids at the polymorphic sites belong to different aa groups, except the aa at position 100, indicated high divergence in the chemical and structural properties of the aa of ORF3 in different PCV2 strains.

**Table 1:** The distribution of amino acids at polymorphic sites in ORF1, ORF2, and ORF3, from 45 PCV2 strains

	Amino acid position	The distribution of amino acids at the polymorphic sites			
ORF1	33	8 D	37 E		
	77	23 F	22 L		
	105	18M	27 I		
	121	4 S	41T		

ORF2	2	14 F	31Y		
	30	39V	6 L		
	53	39F	6I		
	57	34V	12 I		
	59	15R	7K	23A	
	63	25R	11K	5S	4T
	75	39N	6 K		
	76	40I	5L		
	77	37N	8D		
	87	8 T	37 S		
	89	31P	14K		
	90	13R	13I	19L	
	91	22T	13S		
	92	23V	12I		
	134	9N	36T		
	151	28T	17P		
	169	10R	33S	2G	
	190	19T	14S	12A	
	191	5R	13A	27 G	
	206	30I	15K		
	210	13E	14D		
	215	8I	37V		

ORF3	29	34T	11A		
	40	12 R	10S	23G	
	88	41Q	4E		
	100	25F	20L		
	101	9Y	9N	6D	21H
	102	32Q	13K		

## 4. Discussion

In the present study, the diseased piglets were infected with PRRSV and PCV2 viruses, detected by PCR and RT-PCR amplification, therefore, the levels of exposure to the infectious agents were theoretically identical for all animals. The severe lesions and clinical symptoms indicated that PRRSV could be a predisposing factor to the high mortality, as previously suggested [14]. Several hypothesis have been suggested to explain this epidemiological situation, PRRSV may interfere with PCV2 clearance, favor the persistence of the virus. However, the nature of the interaction between PRRSV and PCV2 has not been elucidated to date. The ORF2 protein has been considered as a major immunogenic capsid protein, able to stimulate a protective response in pigs. Five immunoreactive epitopes in ORF2 were identified by Mahe' et al using Pepscan analysis[15], these included residues 25-43, 65-87, 113-147,157-183 and 193-207. Larochelle et al (2002) also identified three major regions of aa heterogeneity among ORF2 sequences at residues 59-80, 121-136 and 180-191, and two of these regions corresponded to two of the immunoreactive epitopes demonstrated by Mahe' et al [16]. In this study, we identified six highly polymorphic regions, 26-50, 57-83, 90-120, 134-154, 169-191 and 206-215, by polymorphic analysis, overlapped with corresponded immunoreactive epitopes, thus demonstrated that polymorphic analysis could be applied to deduce the immunoreactive epitopes of viruses. Our analysis show that ORF1 is highly conserved with only four polymorphic sites, and the amino acids at each of the sites belong to the same groups regarding chemical structure, which indicate that these mutations have no big influence on the activity of the Rep protein. Polymorphic sites were clustered in the capsid protein, and overlapped with the immunoreactive epitopes, So ORF2 may play a major role in the varied pathogenicity of PCV2 isolates.

## Competing interests

The authors declare that they have no competing interests.

## Authors' contributions

JZ carried out the molecular genetic studies, participated in the sequence alignment and drafted the manuscript. CQ carried out the immunoassays and participated in the sequence alignment. JZ participated in the design of the study and performed the statistical analysis. All authors read and approved the final manuscript.
